# Activity Dynamics and Signal Representation in a Striatal Network Model with Distance-Dependent Connectivity

**DOI:** 10.1523/ENEURO.0348-16.2017

**Published:** 2017-08-23

**Authors:** Sebastian Spreizer, Martin Angelhuber, Jyotika Bahuguna, Ad Aertsen, Arvind Kumar

**Affiliations:** 1 Faculty of Biology, University of Freiburg, Freiburg, D-79104, Germany; 2 Bernstein Center Freiburg, University of Freiburg, Freiburg, D-79104, Germany; 3 Institute of Neuroscience and Medicine (INM-6), Institute for Advanced Simulation 4 (IAS-6), Research Centre Jülich, Jülich, D-52428, Germany; 4 Department of Computational Science and Technology School of Computer Science and Communication, KTH Royal Institute of Technology, Stockholm, SE-100 44, Sweden

**Keywords:** Basal ganglia, inhibitory networks, neuronal assembly, Parkinson’s disease, striatum

## Abstract

The striatum is the main input nucleus of the basal ganglia. Characterizing striatal activity dynamics is crucial to understanding mechanisms underlying action selection, initiation, and execution. Here, we studied the effects of spatial network connectivity on the spatiotemporal structure of striatal activity. We show that a striatal network with nonmonotonically changing distance-dependent connectivity (according to a gamma distribution) can exhibit a wide repertoire of spatiotemporal dynamics, ranging from spatially homogeneous, asynchronous-irregular (AI) activity to a state with stable, spatially localized activity bumps, as in “winner-take-all” (WTA) dynamics. Among these regimes, the unstable activity bumps [transition activity (TA)] regime closely resembles the experimentally observed spatiotemporal activity dynamics and neuronal assemblies in the striatum. In contrast, striatal networks with monotonically decreasing distance-dependent connectivity (in a Gaussian fashion) can exhibit only an AI state. Thus, given the observation of spatially compact neuronal clusters in the striatum, our model suggests that recurrent connectivity among striatal projection neurons should vary nonmonotonically. In brain disorders such as Parkinson’s disease, increased cortical inputs and high striatal firing rates are associated with a reduction in stimulus sensitivity. Consistent with this, our model suggests that strong cortical inputs drive the striatum to a WTA state, leading to low stimulus sensitivity and high variability. In contrast, the AI and TA states show high stimulus sensitivity and reliability. Thus, based on these results, we propose that in a healthy state the striatum operates in a AI/TA state and that lack of dopamine pushes it into a WTA state.

## Visual Abstract

**Figure:**
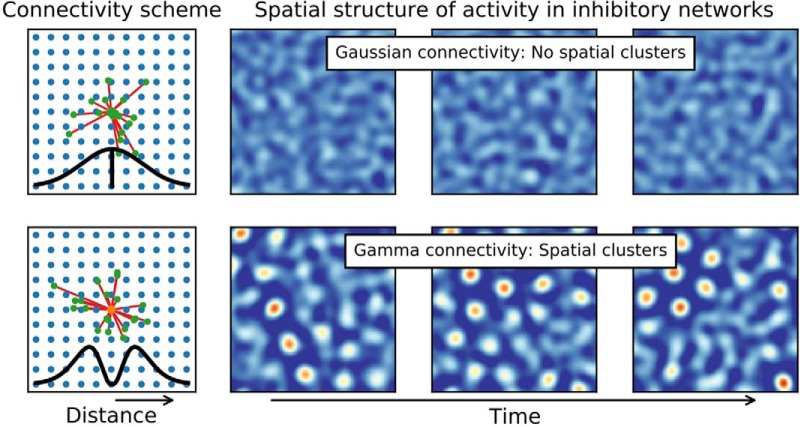

## Significance Statement

Recent findings suggest that striatal activity is organized in spatially compact neuron clusters. Here, we show that striatal projection neurons should have a nonmonotonically changing distance-dependent connectivity to obtain spatially localized activity patterns in striatum. Among the different states a striatal network can show, asynchronous-irregular and transition activity states closely resemble striatal activity in the healthy state. In contrast, strong cortical inputs as observed in Parkinson’s disease drive the network into a winner-take-all state, in which the striatum loses its stimulus sensitivity. Thus, our model makes specific predictions about the spatial network connectivity in the striatum and provides new insights about how the striatum might make a transition from a healthy state to a Parkinson’s disease state.

## Introduction

The basal ganglia (BG) are a collection of nuclei located at the base of the forebrain. BG are crucial for critical functions such as voluntary movement control, decision-making, procedural learning, and a variety of cognitive functions. The striatum as the main input stage of the BG plays a major role in BG-related brain function and dysfunction. Like many other subcortical structures, the striatum is a network of inhibitory neurons driven by excitatory inputs from multiple regions of the neocortex (Wall et al., 2013), thalamus (Smith et al., 2009a), and hippocampus. The output of the striatum directly projects to the globus pallidus (GP) and forms two major functional pathways in the BG, the “go and “no-go” pathways. The interaction of these pathways forms the basis of functions such as action selection, initiation, and execution (Albin et al., 1989).

Experimental data suggest that striatal firing rates in ongoing activity are quite low (≤1 Hz; Mahon et al., 2004; Gage et al., 2010), and ≈30% of striatal projection neurons respond with a markedly increased firing rate (≈10–20 Hz; Kimchi and Laubach, 2009; Gage et al., 2010; Adler et al., 2012). However, it remains unclear how the sparse and low-firing-rate task-related activity of the striatum is organized in space and time to shape the activity of the GP to initiate action selection.

Recent observations of temporal (Adler et al., 2013) and spatial (Barbera et al., 2016) clustering of spiking activity in the striatum suggest that striatal activity may be organized in transient coactivations of groups of neurons. Such coactivated neuron groups can be termed neuronal assemblies (NAs). Such NAs can effectively increase the inhibitory influence of striatal neurons on downstream networks. However, conditions under which NAs appear in the striatum are not well understood.

Within the framework of dynamical systems, the NA-type activity state could be an outcome of a “winnerless competition” (WLC; Rabinovich et al., 2001) or a noise driven transition state between the asynchronous-irregular (AI) activity state and the “winner-take-all” (WTA) state. Although the mechanisms underlying the emergence of such transient NAs are not well understood, heterogeneity in both the external input and the recurrent connectivity among striatal projection neurons is a common feature of the existing models of transient NAs (Humphries et al., 2009; Ponzi and Wickens, 2010; Angulo-Garcia et al., 2015).

Here, we investigate the existence of NAs in a large-scale network model of the striatum in which neurons are connected in a distance-dependent manner. We address two key questions: (1) How does the structure of the network connectivity define the repertoire of the ongoing activity dynamics? and (2) Under which conditions can task-related inputs modify these ongoing activity dynamics?

Specifically, we considered two qualitatively different spatial connectivity profiles in which the connection probability between any two neurons varied as a function of the distance between the neurons: decreased monotonically (according to a Gaussian distribution) or changed nonmonotonically (according to a gamma distribution; Rinzel, 1998). Using network simulations and mathematical analysis, we show that spatially structured activity in the network can emerge only for a nonmonotonically shaped connectivity kernel. In inhibitory networks with a monotonically changing connectivity kernel, network activity remains AI. Only networks with a nonmonotonically changing connectivity kernel can exhibit different activity regimes. Weak background inputs or high input variance induced unstructured AI activity, whereas stronger background inputs or low input variance induced stable dynamics (e.g., WTA). For moderately strong inputs, the network activity organized into unstable spatial bumps [transition activity (TA)] resembling the experimentally observed NAs (Adler et al., 2013; Barbera et al., 2016). Finally, we describe how stimulus response characteristics are affected by the ongoing activity state of the network. We show that only AI and TA states support a reliable stimulus response and that stimulus-induced changes in pairwise correlations are maximal in the TA state, especially for weak stimuli.

These results help us to better understand information processing in the striatum and other primarily inhibitory subcortical networks as in the amygdala and the cerebellum. Moreover, our model provides new insights into neuronal mechanisms underlying brain diseases such as Parkinson’s disease (PD). For instance, the strong resemblance between the properties of the WTA state and the activity observed in PD (Costa et al., 2006) leads to the hypothesis that PD may be a transition from AI or TA to a WTA state.

## Materials and Methods

### Neuron model

Neurons in the network were modeled as “leaky-integrate-and-fire” (LIF) neurons. We intentionally chose this simple neuron model to ensure that the observed network dynamics would be the result of the connectivity profiles studied and not the intrinsic dynamics of a more complex neuron model. The subthreshold membrane potential dynamics of LIF neurons are given by

(1) $$C_{m}\times \frac{dv}{dt}=-g_{L}\times [v(t)-E_{L}]+I(t).$$

Synapses were modeled as conductance transients. The temporal profile of the transient conductance change in response to a single presynaptic spike was modeled as an alpha function. The recurrent connectivity within an inhibitory network such as the striatum is weak and sparse (Tepper et al., 2004; Taverna et al., 2008; Planert et al., 2010). We adjusted the synaptic conductances to obtain weak synapses such that a unitary inhibitory postsynaptic potential (IPSP) had an amplitude of 0.8 mV at a holding potential of –44.0 mV and an excitatory postsynaptic potential (EPSP) had an amplitude of 1.6 mV at a holding potential of –70.0 mV. The various neuron and synapse parameters are summarized in Table 1. Whenever possible, we used parameters corresponding to the striatal network (Yim et al., 2011).

**Table 1.** Parameter values for the neuron and synapse model

Name	Value		Description
**C*_*m*_*	200.0	pF	Membrane capacitance
**g*_*L*_*	12.5	nS	Leak conductance
**E*_*L*_*	–80.0	mV	Leak reversal potential
**V*_*th*_*	–45.0	mV	Spike threshold
**V*_*reset*_*	–80.0	mV	Resting membrane potential
**t*_*ref*_*	2.0	ms	Refractory period
**E*_*exc*_*	0.0	mV	Excitatory reversal potential
**E*_*inh*_*	–64.0	mV	Inhibitory reversal potential
*τ_*exc*_*	5.0	ms	Time constant of excitatory conductance
*τ_*inh*_*	10.0	ms	Time constant of inhibitory conductance

### Network architecture

We considered a population of 10,000 inhibitory neurons. The neurons were placed on a 100 × 100 grid and folded as a torus to avoid boundary effects. The distance between neighboring nodes in the grid network amounted to 10 µm. Each neuron sent 1000 inhibitory recurrent inputs to other neurons within the network (i.e., 10% total connection probability), mimicking the sparse connectivity in most biological inhibitory networks. We implemented no self-connections within the network, and neurons were allowed to form multiple connections between them.

**Figure 1.**
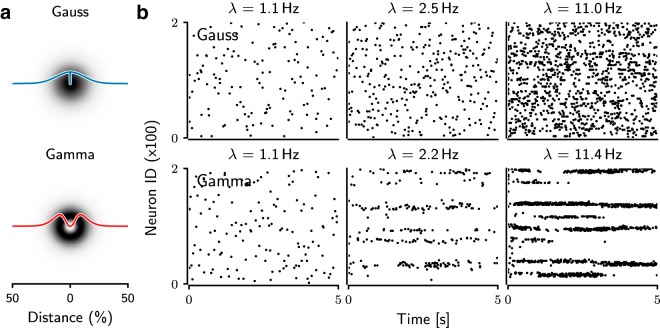
Spiking activity of the network with gamma-distributed (top) and Gaussian-distributed (bottom) connectivity. (a) Connection probability between neurons as a function of the distance between neurons normalized to the full size of the network. (b) Examples of the spiking activity of 200 of 10,000 neurons for different average firing rates ($\bar{\lambda }$) of the network. With increasing background activity, the spiking activity of the Gaussian networks remained irregular and the bursting behavior increased. In contrast, the gamma networks showed transient or persistent bursting behavior and local synchronization of spiking activities.

To implement a distance-dependent connectivity, we chose two qualitatively different spatial profiles (Rinzel, 1998). For the first type of connectivity, we assumed that the distance-dependent connection probability decreased monotonically as a function of distance. To implement such connectivity, we used the Gaussian distribution to model the distance-dependent decrease in connectivity (Fig. 1*a*, top). We will refer to this network type as a Gaussian network.

For the second type of connectivity, we assumed that the distance-dependent connection probability varied nonmonotonically as a function of distance. Recent experimental data suggest that the connectivity between pairs of medium spiny neurons (MSNs) in the striatum may indeed change nonmonotonically as a function of distance (Fujiyama et al., 2011; López-Huerta et al., 2013). To implement such connectivity, we used a gamma distribution to model the distance dependent variation in connectivity (Fig. 1*a*, bottom). We will refer to this network type as a gamma network.

The out-degree of individual neurons was fixed to 1000 neurons. In our network, target neurons were located on a uniformly spaced grid (100 × 100), and each neuron index can be calculated by its coordinate on the grid map. Moreover, each target neuron could also be identified by an angle and radius in polar coordinates:

$$\begin{array}{c} x=r\times sin(\phi )\\ y=r\times cos(\phi )\\ idx=x\times ncol+y \end{array}$$

where *x* and *y* are the coordinates of the grid locations, *idx* is the index of the target neurons, and *ncol* = 100 is the size of the network in one of two dimensions. To identify 1000 targets, we drew ϕ from a uniform distribution U(−π,π) and *r* from either Gaussian or gamma distribution. This approach is much faster than deciding connectivity based on pairwise distances, but can be used only when neurons are arranged on a grid.

To appropriately compare these different network types, we ensured that the connection weights and the degree distributions were identical in the two network types.

### Ongoing/background excitatory drive

All neurons received background excitatory inputs mimicking the global ongoing activity in striatum. The external drive was modeled by uncorrelated homogeneous Poisson-type spike trains with fixed firing rate. Each neuron received an independent realization of such spike train, obtained from the same underlying Poisson process. The rate of the Poisson-type spike trains was varied systematically to study the different dynamical states of the inhibitory networks.

### Stimulus

To measure the impact of the an external stimulus on network activity in each dynamic state, we stimulated the network with an external stimulus, on top of the background inputs described above. The stimulus was provided to a subset of ≈45 neurons. To distribute the stimulated neurons in a spatial manner, we defined a region of interest (ROI) of size 30 × 30 neurons. In the ROI, we defined a location as center for stimulated neurons chosen according to a two-dimensional (2D) Gaussian profile (σ: 2 grid points). The external stimulus was modeled as an injection of direct current to the selected neurons. The stimulus presentation lasted for 1 s, and its amplitude was systematically varied between 0 and 150 pA. To collect sufficient data for further statistical analysis, each stimulus was presented 20 times to its assigned subset of neurons.

To estimate how the response magnitude and trial-by-trial variability changes when one stimulus is immediately followed by another, we extended the simulation of the network with two different external stimuli. Each of these two external stimuli was provided to two nonoverlapping subsets of ≈45 neurons. In the ROI, as described above, we defined two locations that were 10 grid points apart. Using these two locations as centers, stimulated neurons were chosen according to a 2D Gaussian profile (σ: 2 grid points). The two stimuli were presented in alternating fashion, and each stimulus presentation lasted for 1 s, separated by a pause of 2 s. Thus, the two stimuli differed only in terms of their target neurons; other properties (strength, duration) were identical.

### Characterization of the network dynamics

The network activity states were characterized on the basis of the spiking activity using standard descriptors: firing rates and the coefficient of variation of interspike intervals (*CV_ISI_*), the latter used to measure the degree of irregularity of the spiking of individual neurons Kumar et al. (2008b).

### Count and duration of activity bumps

We constructed a series of binary matrices representing the 2D map (100 × 100 neurons) of the network activity over disjunct time windows of 100 ms each from the spike train recordings of all neurons. To enhance the spatial contrast, these matrices were convolved with the 2D “Mexican hat”–shaped kernel (Ricker wavelet). The size of the hat was taken from the estimation of the bump size (Figs. 4*b* and 5). The integral of the Mexican hat kernel was set to zero to ensure that the filtered activity was comparable for different inputs. Additionally, the integrals of the positive and the negative part of the convolution kernel were set to 1 and –1, respectively. After convolving the spatial activity of the network (measured in a 100 ms window) with the Mexican hat kernel, we thresholded the resulting image to identify patches of high activity. The center of each patch was taken as the position of individual bumps. The positions of individual bumps were then used to compute the number of bumps and determine the affiliation of neurons to individual bumps. Finally, trackers of time-merged bumps were used to analyze the lifespan of the bumps, determined by finding similar positions of bump centroids over subsequent time frames.

### Characterization of the stimulus response

To estimate the influence of the external stimuli on striatal ongoing activity, we analyzed the activity of stimulated neurons in the ROI (of size 30 × 30 grid). To characterize the impact of the external stimulus on the network activity dynamics, we measured the average firing rate, the trial-by-trial variability of the network response, and the spectrum of pairwise correlations of the network spiking activity. To estimate whether the evoked response of the stimulated neurons was discernible from the background, we measured

$$\Delta \text{response}=\frac{\text{evoked}\;\text{firing}\;\text{rate}}{\text{background}\;\text{firing}\;\text{rate}}$$

for each stimulus. The trial-by-trial variability was quantified using the Fano factor (FF), defined as the ratio of the variance and the mean of the response of the stimulated neurons.

The correlation spectrum was estimated for the activity of individual neurons. Neuronal spiking activity was binned using a rectangular bin of 100 ms. We measured the correlation spectrum for the trial-by-trial average of both ongoing and stimulus-evoked activity.

### Simulation tools

All simulations of the networks of spiking neurons were performed using NEST simulation software (Gewaltig and Diesmann, 2007). The dynamical equations were integrated at a fixed temporal resolution of 0.1 ms. Simulation data were analyzed with Python using the scientific libraries SciPy (http://www.scipy.org) and NumPy (http://www.numpy.org) and visualized using the plotting library Matplotlib (http://matplotlib.org).

## Results

Neuronal activity in the striatum is maintained by thalamic and cortical excitatory inputs. In a randomly connected inhibitory network model of the striatum, the recurrent inhibition and the level of external excitatory inputs define the dynamical states and stimulus response properties of the network (Brunel and Hakim, 1999; Ponzi and Wickens, 2010; Yim et al., 2011; Angulo-Garcia et al., 2015). Here, we extend this earlier work by investigating the effects of spatial connectivity on the dynamical states and the stimulus-response properties of the striatal network activity. To this end, we used both analytical calculations using neural field equations and numerical simulations of biologically realistic, large-scale inhibitory networks models with 10,000 spiking neurons.

In our network models, the profile of the spatial connection probability between any two neurons could vary either monotonically or nonmonotonically as a function of the distance between the neurons. For this, we used two different kernels for the spatial connectivity: In Gamma networks, the nonmonotonic distance-dependent change in connection probability was modeled as a Gamma distribution [off-center inhibition (Rinzel, 1998); Fig. 1*a*, top], whereas in Gaussian networks, the connection probability decreased monotonically in a Gaussian manner as a function of distance between neurons [on-center inhibition (Rinzel, 1998); Fig. 1*a*, bottom].

### Firing rates and spike train irregularity

**Figure 2.**
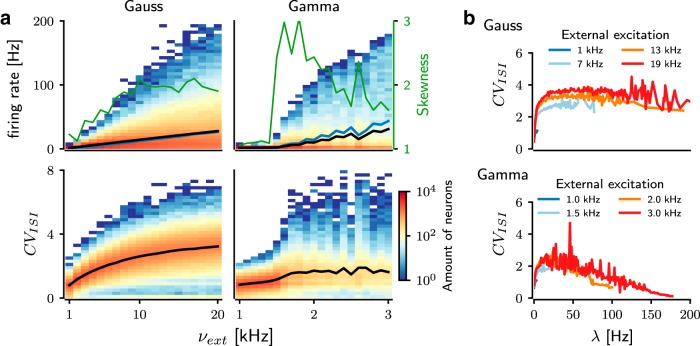
Analysis of the spike patterns in networks with different connectivity profiles. ***a***, Distribution of average firing rates ($\bar{\lambda }$; top) and coefficient of variation (*CV_ISI_*; bottom) as a function of background input strength (*ν_ext_*) for Gaussian (left) and gamma (right) networks. In Gaussian networks, increasing *ν_ext_* resulted in a steadily widening distribution of $\bar{\lambda }$ and *CV_ISI_*, which for a large fraction of neurons tended to the value 3. In contrast, in gamma networks, the distribution of $\bar{\lambda }$ and *CV_ISI_* was rapidly widely distributed from a relatively low *ν_ext_* (1.5 kHz) upward. Gamma networks were clearly more excitable than Gaussian networks. Green trace indicates the skewness of the firing rate in both networks. Black and blue traces refer to the average firing rate ($\bar{\lambda }$) and standard deviation (σ_λ_) of the firing rate distribution, respectively. ***b***, Relationship between the irregularity of the spiking pattern (*CV_ISI_*) and the average firing rate ($\bar{\lambda }$). The color of the traces represents the background input rate (*ν_ext_*). In Gaussian networks (top), neurons with higher $\bar{\lambda }$ tended to exhibit a higher *CV_ISI_*, whereas in gamma networks (bottom), they tended to exhibit a lower *CV_ISI_*.

First, we determined the firing rate and the degree of irregularity of the spiking activities in the networks connected according to the Gaussian-shaped (Figs. 1*a*, *b*, top) and gamma-shaped (Figs. 1*a*, *b*, bottom) connectivity profiles. As expected, the average firing rates in both network types increased as a function of the background excitatory input (*ν_ext_*; Fig. 1*b*). However, comparing the network activity at similar average output rate ($\overset{¯}{\lambda }$), the activity pattern of network differed in connectivity profiles (Fig. 1*b*). Gamma networks were clearly more sensitive to a change in the background input rate (*ν_ext_*) than Gaussian networks (Fig. 2*a*, black trace). Thus, the same range of average firing rates as in Gaussian networks was achieved in gamma networks already for a much lower range of external inputs (*ν_ext_*; Fig. 2*a*, black trace). This suggests that a gamma-shaped spatial connectivity kernel increased the sensitivity of the network activity to changes in the background input.

Moreover, these two network types differed considerably in the distribution of firing rates over neurons (Fig. 2*a*, top row) and spike timing irregularity (*CV_ISI_*; Fig. 2*a*, bottom row). We compared the output firing rate distributions for a range of external inputs that resulted in the same range of output firing rates. The recurrent inhibition in the network ensured that even for very strong background input, only few neurons could spike at a high rate, and a major fraction of neurons spiked at relatively low rates, resulting in a positively skewed distribution of firing rates. For Gaussian networks, the average firing rate ($\bar{\lambda }$) and the standard deviation (σ_λ_) and skewness (κ_λ_) of the neuronal firing rate distribution monotonically increased as background input was increased (Fig. 2*a*, top right). In contrast, in Gamma networks, both $\bar{\lambda }$ and σ_λ_ increased nonmonotonically, but the skewness of the firing rate distribution changed in a nonmonotonic fashion as a function of background input rate (Fig. 2*a*, top right). This suggests that a nonmonotonically changing connectivity profile results in a larger diversity of firing rates in the network and that the diversity of firing rates is maximized for a moderate amount of background input.

Interestingly, in Gaussian networks, the neurons’ *CV_ISI_* monotonically increased with an increase in their firing rate. An average *CV_ISI_* > 1 indicates that most high-firing-rate neurons spiked in bursts (Fig. 2*b*, top). Because we used LIF neurons, the bursting in the network activity was caused by the recurrent inhibition and not intrinsic neuron properties. In the Gaussian networks, the connection probability peaked over only a small range in the vicinity of a given neuron, thereby reducing the effective recurrent inhibition and allowing the neuron to maintain its high firing rate for some time. However, fluctuations in the external input (modeled as Poisson spike trains) could rapidly switch the high-rate activity from any one neuron to another, thereby creating a spike pattern consisting of short-lived bursts and pauses.

In contrast, in the Gamma networks, only neurons with moderate output firing rates showed a *CV_ISI_* > 1 (Fig. 2*b*, top). Neurons with very high firing rates ($\bar{\lambda }$ ≥100 Hz) spiked in a regular manner (*CV_ISI_* ≤0.5). Such a small *CV_ISI_* implies that the network was operating in the so-called mean-driven regime (Brunel, 2000), in which the background input is strong enough to keep the free-membrane potential of the neurons above spiking threshold. Within the physiologic range of output firing rates ($\bar{\lambda }$ ≤50 Hz), neurons in both network types elicited spike bursts, but the *CV_ISI_* in Gaussian networks was nearly twice as high as that in Gamma networks.

### Spatial structure of the network activity

**Figure 3.**
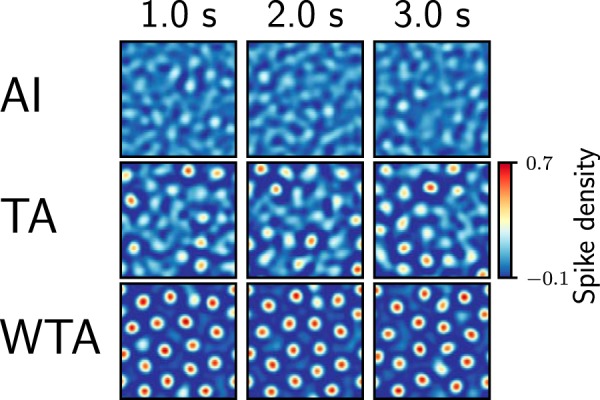
Characterization of the bump activity states for the gamma network. Time series snapshots of the 2D pattern (100 × 100 neurons) of bump activity, contrast-enhanced by Mexican hat filtering. Each frame was measured by summing the neuronal activity over 100 ms. Three snapshots (columns) of activity were taken 1 s apart. Three representative gamma network states for three different amounts of external inputs are shown in each row. AI: external input = 1 kHz; no bump activity is observed and the network activity remains noisy. TA: external input = 1.5 kHz; the network is in an unstable state, with several bumps appearing transiently, in the company of noisy activity. WTA: external input = 3 kHz; the network forms mostly persistent bumps throughout the entire network.

**Figure 4.**
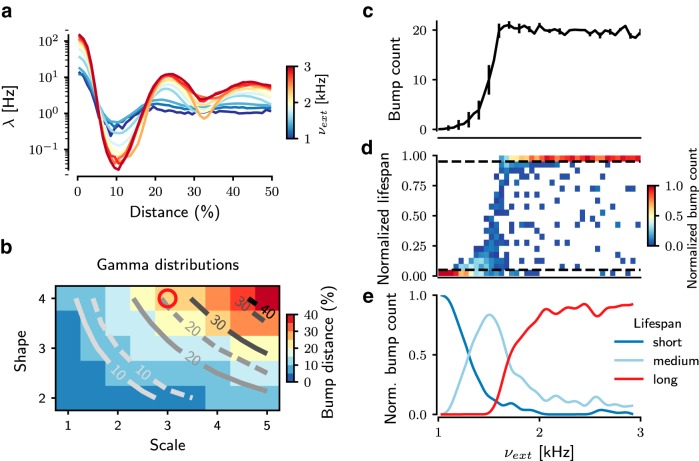
Quantification of bump activities in gamma networks. ***a***, Spatial autocorrelation of the network spiking activity, showing the mean firing rate ($\bar{\lambda }$) of each bump as a function of the distance from the cluster centroid normalized to the full size of the network. Different colors represent the strength of the background excitation. ***b***, Distance between bumps in various gamma distributions and its comparison between numerical simulations and mean field equations. The background color is measured bump distance from the network simulation data. Solid traces show results from analytical estimation of bump distance; dashed traces represent the estimation of bump distance from network simulations. The parameter for the gamma-distributed connection is used for the spiking network model (red circle). Spatial representations of activity bumps are also observed in Fig. 5 (*c–e*). These subfigures display the bump count and their relative lifespan during the entire simulation as a function of the background external excitation (*ν_ext_*), which modulates the nature of the bump activity. ***c***, With increasing *ν_ext_*, the number of bumps increases in a sigmoidal fashion. For higher *ν_ext_*, the number of bumps saturates, owing to the limited capacity of the finite spatial map. The error bars for the bump counts indicate the standard deviation of bump counts over the simulation time. ***d***, The lifespan of bumps reflects the dynamic state of the bumps: a shorter lifespan reflects TA dynamics, whereas a larger lifespan indicates stable bump activity reflecting WTA dynamics. By increasing *ν_ext_*, the distribution of lifespans shifts from short to long term. The ordinate indicates lifespan, normalized to the duration of the entire simulation (10 s), of individual bumps. Because the average bump counts are different in each dynamical state (TA, WTA), we normalized the color bar of bump count to the average bump count in individual states. ***e***, The lifespan distribution is split into three groups (dashed lines in ***d***). The long (red trace) appearance of bumps reflects the WTA state of bump activity, whereas the short (blue trace) appearance of bumps reflects the TA state of bump activity. Between these two states, the network is in a highly unstable state, characterized by a wider distribution of lifespans (light blue trace).

Next, we included spatial information about neurons in our analysis and characterized the spatial activity patterns in both network types. Visual inspection of the spike rasters (Fig. 1*b*) suggested that in Gaussian networks, the structure of the network activity remained spatially homogeneous, even for very high background input rates (*ν_ext_*). In contrast, when increasing the background input rate in gamma networks, the activity got confined into local clusters, resulting in spatially periodic activity bumps. The spatial structure became more apparent when we rendered the neuronal activity as a 2D surface (Figs. 3 and 4*a*). Depending on the strength of the background input, three qualitatively different network activity states were observed. In the AI state, neurons spiked at a low rate and the activity was more or less homogeneously distributed across the network (Fig. 3, top row). For very strong background inputs, the network activity organized into a spatially periodic and temporally stable bump structure (Fig. 3, bottom row). This state resembles the *k*-WTA state (Hutt and Atay, 2005). In between, for moderate inputs, the bump structure was aperiodic and unstable (Fig. 3, middle row). We refer to this state as transition activity (TA).

To better characterize the emergence of activity bumps and these three dynamic states in gamma networks, we measured the numbers, sizes, and lifespans of activity bumps. To reliably identify bumps, we designed a bump detection algorithm (see Methods). First, we measured the average firing rate as a function of distance from the center of a bump. For weak inputs, the average network activity decayed and reached a baseline level with increasing distance from the center of the bump. However, for stronger inputs, not only did the firing rate in the bump increase, but the spatially periodic nature of the activity also became more apparent (Fig. 4*a*).

The number of bumps increased in a sigmoidal fashion with the background input rate (Fig. 4*c*). For strong inputs (*ν_ext_* ≥1.7 Kspikes/s), the bumps were stable: neurons that started to spike at the beginning of the simulation remained active for the entire simulation (Fig. 4*d*, *e*, red trace). For weak inputs (*ν_ext_* ≤1.2 Kspikes/s), the few bumps that appeared lasted only for short durations (Fig. 4*d*, *e*, blue trace). For the medium range of input rates (1.2 ≤ *ν_ext_* ≤ 1.7 Kspikes/s), the bumps showed a wide range of lifespans (Fig. 4*d*, *e*, light blue trace).

This analysis of the network activity showed that gamma networks exhibited random unstructured activity for weak background inputs and stable periodic bump activity for strong background inputs. The stable bump activity was similar to the periodic bump activity observed in networks of excitatory and inhibitory neurons interconnected according to a spatial Gaussian connectivity profile (Mehring et al., 2003; Roxin et al., 2005; Kumar et al., 2008a). This raises the question of why inhibitory networks with a gamma-type connectivity profile exhibit spatial bump activity patterns and why networks with Gaussian-distributed connectivity profile do not.

### Necessary conditions for the emergence of bump states

To understand why a gamma-type connectivity profile exhibits spatial bump activity and a Gaussian connectivity profile does not, we investigated the dynamical states of spatially interconnected inhibitory neurons using neural field equations (Ermentrout, 1992; Hutt and Atay, 2005). For simplicity, we started by formulating the neural field equation for a one-dimensional network with circular boundary conditions. The results hold also for a 2D network with torus-folded boundary conditions because we are considering a homogeneous and isotropic scenario. The mean membrane potential *v*(*x*,*t*) in the continuum limit is given by

(2) $$\frac{\partial v\left(x,t\right)}{\partial t}=-v\left(x,t\right)-\int_{-\infty }^{\infty }f\left(v\left(y,t\right)\right)W\left(x-y\right)dy+I\left(x,t\right),$$

where *W*(*x*) denotes the spatial connectivity profile and *f*(*v*) the neuron transfer function, mapping the mean membrane potential to the output firing rate ($\bar{\lambda }$). Without loss of generality, we used normalized connectivity profiles *W*(*x*), and the scaling parameter was chosen such that the absolute connection strength was absorbed into *f*(*v*). The background input to the neurons is denoted by *I*(*x*,*t*). For the network, the stationary and spatially homogeneous solution *v*(*x*,*t*) = *v*_0_ = constant is a solution to Eq. (2) for a constant background input *I*(*x*,*t*) = *I*_0_. Here, *v*_0_ is given by the implicit equation

(3) $$\frac{\partial v_{0}}{\partial t}=-v_{0}-f(v_{0})+I_{0}=0.$$

To investigate the stability of this homogeneous solution, we considered small perturbations $v\left(x,t\right)=v_{0}+\varepsilon \nu \left(x,t\right)$ around *v*_0_ and linearized the transfer function in Eq. (2). After subtracting Eq. (3), this yields

(4) $$\frac{\partial \nu \left(x,t\right)}{\partial t}\approx -\nu \left(x,t\right)-\int_{-\infty }^{\infty }f\prime \left(v_{0}\right)\nu \left(x,t\right)W\left(x-y\right)dy.$$

Here and in the remainder $f\prime \left(v_{0}\right)$ is used as shorthand notation for $\frac{\partial f\left(v\right)}{\partial v}{|}_{v=v_{0}}$. In the Fourier domain, this expression simplifies to

(5) $$\frac{\partial \overset{˜}{\nu }\left(k,t\right)}{\partial t}\approx -\overset{˜}{v}\left(k,t\right)-f\prime \left(v_{0}\right)\overset{˜}{\nu }\left(k,t\right)\overset{˜}{W}\left(k\right),$$

with $\overset{˜}{\nu }$ denoting the Fourier transform of ν with respect to space. We can now obtain the eigenvalues:

(6) $$\zeta =-1-f\prime \left(v_{0}\right)\overset{˜}{W}\left(k\right).$$

When the eigenvalues are positive, small perturbations do not die out, indicating unstable dynamics. Assuming that the slope of the transfer function $f\prime \left(v_{0}\right)$ is always nonnegative, negative values in $\tilde{W}\left(k\right)$ are a necessary condition for positive eigenvalues ζ and, hence, for spatially periodic activity bumps. In purely inhibitory networks, this condition can be fulfilled by off-center inhibition kernels, such as the gamma-kernel under investigation, or, e.g., a mixture of two Gaussian distributions arranged symmetrically around zero. Although for biologically plausible connection kernels, off-center inhibition (i.e., a nonmonotonic kernel) fulfills this condition, it should be noted that this is not a necessary condition. For instance, the Fourier transform of a box-shaped kernel around zero takes negative values at nonzero frequencies and therefore could, in principle, generate bump states.

For the gamma distribution–shaped connectivity kernel,

(7) $$W\left(x\right)=\frac{x^{n-1}e^{\frac{-|x|}{\Theta }}}{2\Gamma \left(n\right)\Theta^{n}},$$

with shape parameter *n* and scale parameter Θ, this condition of positive eigenvalue ζ > 0 can be fulfilled for *n* > 1, when the Fourier transform $\tilde{W}\left(k\right)$ takes negative values (Fig. 6, right). In contrast, the Gaussian kernel Fourier-transforms into another Gaussian, which never takes negative values (Fig. 6, right), and therefore, purely inhibitory networks with a Gaussian-type connectivity profile do not show any spatially periodic bump activity. When the eigenvalues *ζ* are negative, the network activity remains spatially homogeneous, similar to the AI state observed in both the Gaussian networks and the gamma networks (Fig. 1*c*).

Eq. (6) also revealed that the slope of the transfer function at stationary rate $f\prime \left(v_{0}\right)$ is an important factor controlling the eigenvalues. For positive eigenvalues, it needs to be sufficiently large. That is, the following condition needs to be fulfilled:

(8) $$f\prime \left(v_{0}\right)>{\left[\underset{{}_{k}}{\min}\overset{˜}{W}\left(k\right)\right]}^{-1}.$$

Here, $f\prime \left(v_{0}\right)$ is controlled by a number of factors. For instance, an increase in the synaptic weights (e.g., cortico-striatal, MSNs to MSNs, and fast spiking interneurons to MSNs) increases $f\prime \left(v_{0}\right)$ and can make the network cross the bifurcation. Similarly, increasing the background input rate will also increase the slope $f\prime \left(v_{0}\right)$, because the transfer function of LIF neurons in the simulated network is convex for low firing rates (Burkitt, 2006). That is, both an increase in the strength of recurrent synapses and an increase in the background input rate can cause the transition from spatially homogeneous firing to periodically organized bump solutions.

This analysis shows that the activity of an inhibitory network driven by constant input *I*(*x*) has two stable solutions: for weak inputs, the stationary state is spatially homogeneous (AI state; Fig. 3, top row), whereas for strong inputs, the stationary state is spatially periodic (WTA state; Fig. 3, bottom row). When the network is driven with noisy inputs or the neurons have unequal numbers of synapses (owing to random connectivity), the transition between the two stable solutions is smoothened (TA state; Fig. 3, middle row). This result of the analytical calculations is consistent with the numerical network simulations (Figs. 1 and 4) where noise was introduced into the network by the connectivity and the Poisson type spike trains as external background input.

In addition to stating the condition for spatially periodic bump solutions to arise, Eq. (6) enables us to estimate the distance between bumps. The wave number of the emerging spatially periodic solution is approximately given by the wavenumber *k_c_* minimizing the Fourier transform of the gamma kernel (7). This critical wavenumber *k_c_* is given by

(9) $$k_{c}=\arg\underset{{}_{k}}{\min}\overset{˜}{W}\left(k\right)=\frac{\tan\frac{\pi }{n+1}}{\Theta }.$$

**Figure 5.**
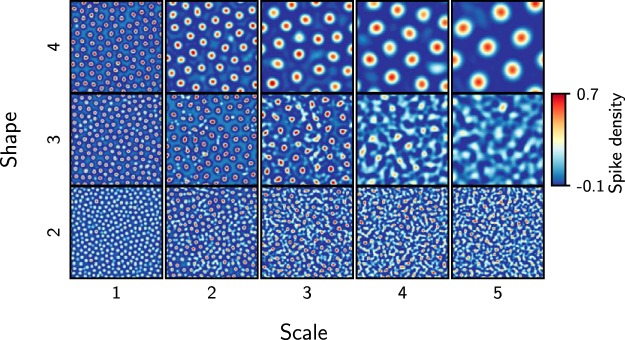
Spatial map of bump activity patterns for different gamma distributions. A snapshot, contrast-enhanced by Mexican hat filtering, of the 2D pattern (100 × 100 neurons) of bump activity for different parameters (shape, scale) of gamma connection profile defines the size of bumps and the distance between bumps. The rate of the external Poisson excitation (*ν_ext_*) was set to 5 kHz to obtain WTA states in networks with different gamma distributions.

This means that the spacing between bumps will increase as the shape (*n*) and scale parameter (Θ) of the gamma function are increased (Fig. 4*b*, dashed traces). We confirmed this in numerical simulations by systematically varying the two parameters of the connectivity kernel (Fig. 5). We found that, indeed, the analytical approximation closely matched the estimates of the interbump distance measured in network simulations (Fig. 4*b*).

### Impact of the ongoing activity state on the stimulus response

The three different networks states we identified were obtained by changing the global input to the network, and therefore, these states can be thought as possible ongoing states of the network activity, i.e., when the animal is not engaged in a specific task. To understand how a task-related activity may be represented in the striatum when it operates in one of these three ongoing activity states, we selectively stimulated two different subsets of neurons located in spatially nonoverlapping regions in the network alternately (stimulus *A* and *B*; Fig. 7*a*; see Methods) and measured the stimulus response.

In the AI state, the network instantaneously responded to the stimulus and switched to a different spatial pattern as the input was changed (Fig. 7*a*, top row). In this state, there were no bumps in the ongoing activity; therefore the stimulus created new bumps. In the TA as the ongoing state (Fig. 7*a*, middle row), external stimuli modified the already existing bump pattern. The resulting patterns were different for the two stimuli. By contrast, in the WTA state, the ongoing activity already showed strong stable bumps and the external stimuli proved insufficient to alter the ongoing bump pattern (Fig. 7*a*, bottom row), unless it overlapped with an existing bump (e.g., the stimulus *B* in Fig. 7*a*, bottom row).

To quantify the striatal response in different ongoing activity states, we measured the change in the average firing rate of the stimulated neurons (Δresponse, Fig. 7*b*) and the trial-by-trial variability (measured over 20 trials) for the two stimuli (Fig. 7*c*). In AI and TA states, the external input elicited a strong response, well discernible from the background activity. As expected, the response magnitude increased with an increase in the stimulus strength (Fig. 7*b*). The variance of the evoked response (Δresponse measured across trials for the whole duration of the stimulus) in the TA state was higher than in the AI state, but for stronger stimuli both average response and trial-by-trial variability were similar (Fig. 7*b*). The time-resolved trial-by-trial variability of the evoked response (measured as FF) of the network response was maximal at stimulus onset (Fig. 7*c*). However, the trial-by-trial variability of the response in the steady state (in the presence of the stimulus) was smaller than that observed in the ongoing state. By contrast, in the WTA state, not only the Δresponse was smallest among the three states, it was also more variable across trials (depending on the location of the stimulus). For strong input it was possible to elicit a strong reliable response even in the WTA state, but that response depended strongly on the location of the stimulus, e.g., among the two stimuli we tested, only stimulus *B* resulted in a high Δresponse (Fig. 7*b*, bottom row). Besides, in the WTA state, trial-by-trial variability also depended on the stimulus location. For the stimulus A, FF in the WTA state was highest of the three states (Fig. 7*c*). Although average FF of stimulus B was much lower (≈7) than that of the stimulus A (≈25) in the WTA state, it was still higher than that measured in AI and TA states. These observations suggest that evoked responses are far more variable across trials in the WTA state than in both AI and TA states.

Taken together, the stimulus response properties of the three ongoing network activity states in gamma networks showed that only AI and TA states provide a suitable substrate to reliably encode different external stimuli. In contrast, in the WTA state, the response depends not only on the stimulus amplitude but also on the spatial location of the input. When the input coincides with an existing bump, the response is reliable and discernible from the background, but otherwise the response is weak and unreliable (e.g., stimulus *B*).

### Modulation of pairwise correlations is maximal in the TA state

AI and TA states are similar in terms of stimulus sensitivity and response reliability (Fig. 7). Further analysis of the evoked activity, however, revealed a crucial difference between these two network states that renders the TA state as a more suitable ongoing activity state for stimulus encoding. In the AI state, a weak external stimulus affects only the rate of stimulated neurons and, thereby, induces only a small effect on neighboring neurons. Therefore, the correlation spectrum during the evoked activity is slightly positively skewed (Fig. 8, left, light blue trace). That is, although weak stimuli can evoke activity responses in the AI state, the spectrum of correlations remains largely unaffected (Fig. 8).

In contrast, in the TA state, even a weak external stimulus can create new activity bumps and, thereby, introduce both positive and negative correlations (Fig. 8, middle, light blue trace). The resulting distribution of correlations in the evoked activity is much wider than observed in both AI and WTA states. That is, in the TA state even weak stimuli are able to induce large changes in the structure of pairwise correlations (Fig. 8, middle, light blue trace). These correlations may be effective in carving out the selected action not only in the striatum, but also downstream in external and internal GP, both of which have high baseline activity and require coordinated striatal inhibition to be suppressed. Moreover, the change in the pairwise correlation distribution can also be useful in distinguishing stimulus-evoked bumps from spontaneously generated bumps, which otherwise cannot be distinguished based on firing rates alone.

For strong external stimuli, the correlation spectra are similar in both AI and TA states. In the WTA state, however, a weak stimulus did not induce any visible modulation in the correlation spectrum (Fig. 8, right, blue and light blue traces), and only very strong inputs could trigger a small change in the correlation structure (Fig. 8, right, red and orange traces). These results suggest that although both AI and TA states allow for reliable and discernible stimulus responses, the TA state may be more suitable to process weaker stimuli than the AI state, because in the TA state the correlation spectrum can already be modulated by very weak stimuli.

## Discussion

Here, we investigated the activity dynamics and stimulus response properties of the striatum as a purely inhibitory network with different spatial connectivity profiles. We showed that a nonmonotonically changing spatial connectivity profile can lead to the emergence of spatially structured activity in purely inhibitory networks. In contrast, when the connectivity changes monotonically as a function of distance between neurons, the network activity is uniformly distributed over the network, respective of the background input. Specifically, we have shown that with a nonmonotonically shaped connectivity profile, the striatal network can exhibit three qualitatively different activity states: AI, TA, and WTA dynamics. Importantly, among these three different dynamical network states, both AI and TA states have the necessary properties for reliably encoding external stimuli. Between the AI and TA states, the average stimulus response is similar; however, the TA state has several interesting properties that make it a dynamically richer and more responsive state: (1) the overall firing rate distribution in the TA state is more skewed than in the other two states (Fig. 2*a*); (2) the lifespan of NAs in the TA state is more widely distributed that in the other two states (Fig. 4*d*); and (3) in the TA state, even weak stimuli are able to alter the spectrum of pairwise correlations (Fig. 8).

Transient NAs in striatum-like purely inhibitory networks with spatial connectivity structure (Humphries et al., 2009) or without any spatial connectivity structure (Ponzi and Wickens, 2010) have been defined as groups of neurons showing a conspicuous correlation in their temporal firing rate profiles. Such NAs and their member neurons were identified by offline analysis of the spiking activities of neurons in sparsely connected, random recurrent inhibitory network models with weak synapses. In such networks, NAs were found to be randomly distributed over the entire network and appeared to involve mutually unconnected neurons (Angulo-Garcia et al., 2015). However, such assemblies may not influence the downstream network, unless they are specifically wired to share their downstream targets. Moreover, experimental data showed that transient NAs exist as spatially compact clusters (Barbera et al., 2016). Here, we extend the previous work and show that the existence of spatially compact NAs in a striatum-like network requires that the connection probability between striatal neurons changes in a nonmonotonic fashion as a function of their distance.

Previously, a few studies have investigated the dynamics of the striatal network with distance-dependent connectivity (Wickens et al., 1995; Humphries et al., 2010), albeit without focusing on the role of the connectivity structure and the external input. Wickens et al. (1995) showed that when the connectivity of striatal neurons is confined to a small neighborhood, the network can exhibit multiple spatially localized, persistent bumps (akin to the WTA state). Such multiple bump–state activity required that the connections were symmetric. Any heterogeneity and asymmetry in connections led to traveling waves or AI-type activity. However, that network was very small, and the connection probability was fixed over a finite distance. Humphries et al. (2010) developed a more realistic model of the striatum using distance-dependent connectivity estimated from the 3D morphology of MSNs. Structurally, that network is similar to the gamma network we investigated here. However, Humphries et al. (2010) investigated neither the dynamics of the network as a function of the input nor the relationship between network structure and dynamics. Our work complements and extends these previous studies and, importantly, explains how external input and the structure of spatial connectivity profiles (Gaussian and gamma) interact to shape the dynamics of spatially compact activity clusters in the striatum.

### Relevance for striatal network activity dynamics

In the ongoing network activity state, striatal neurons are relatively silent, and task-related activity can increase up to 20 Hz (Gage et al., 2010). Recently, advances in recording methods have enabled recording 10–100 neurons simultaneously, using calcium imaging in behaving animals. Analyses of such high-density sampling of striatal neurons showed that striatal activity is organized as spatially compact clusters of coactivated neurons (or NAs; Barbera et al., 2016; R. Costa, personal communication). Similar observations were made earlier in *in vitro* (Carrillo-Reid et al., 2011) and in task-related activity in behaving monkeys (Adler et al., 2013). In an unhealthy low-dopamine state (as in Parkinson’s disease), D2 type dopamine receptor expressing striatal projection neurons increase their firing rates (Mallet et al., 2006), and in general, striatal neuronal activity loses its diversity and ability to switch neuronal activity in a task-dependent fashion (Costa et al., 2006; Costa, 2011).

It is conceivable that the spatially compact clusters (or NAs) are a simple consequence of spatially localized cortico-thalamic inputs to the striatum. However, this is a trivial solution and indicates that the striatum acts as a simple transmitter of cortico-thalamic activity to downstream targets. Instead here we argue that intrinsic dynamics of striatum-like inhibitory networks are able to generate NAs even when cortico-thalamic inputs are not spatially compact.

The three network states that we identified in our study capture different aspects of the ongoing and evoked activity of the striatum in normal and in low-dopamine states. Both AI and TA states in our network models match some properties of the ongoing activity measured *in vivo*. In data from experiments in which animals wait for a cue to initiate a task (e.g., Gage et al., 2010; Adler et al., 2012), the ongoing activity in the striatum appears be similar to the AI state. When the animal engages in the task, after the cue presentation, the AI activity is transformed into a TA-like state (Gage et al., 2010; Adler et al., 2012). On the other hand, in data from freely moving animals, although the animals are not performing any goal-directed behavior, striatal activity shows spatially compact clusters of coactivated neurons, similar to the TA state (Barbera et al., 2016). Whether the striatal activity recorded in freely moving animals can be treated as an ongoing activity, a goal-directed activity, or a combination of both is an open question. We hypothesize that because animals were not engaged in specific goal-directed behavior, the data reported in Barbera et al. (2016) represent an ongoing activity state in the sense that any task-related cue or reward was absent. It will be interesting to explore whether these spatial activity clusters also emerge in a goal/cue-directed task, thereby possibly hinting at differences in the contextual input received by the striatum during ongoing and goal-directed states.

Thus, in our opinion, depending on the context (e.g., freely moving or cued goal-directed behavior), the ongoing activity of the striatum can be in an AI or TA state. As stated earlier, both states allow for a reliable and discernible response in the striatum. A subtle, but important, difference between the AI and TA states is that in TA-type ongoing activity, even weak stimuli can induce a large change in stimulus-induced correlations. Assuming that neuronal correlations form the basis of modification of synaptic strengths, we speculate that when an animal operates in a TA state, even weak stimuli can drive learning.

In the WTA state, neurons have higher firing rates and the spatial bumps are stable (Figs. 3 and 4). In this state, the ongoing activity shows only a very small diversity, and only very strong inputs, arriving in specific locations, can induce any discernible and reliable response. With these properties, the WTA state resembles the striatum dynamics in Parkinson’s disease (Costa et al., 2006; Costa, 2011). Moreover, the WTA state is observed when neurons are spiking at a high rate (Fig. 4), which could be achieved either by increasing the ongoing external excitation or by increasing the excitability of the neurons. This is also consistent with the fact that in Parkinson’s disease the cortical drive to the striatum is increased due to the potentiation of cortico-striatal synapses (Smith et al., 2009b; Fieblinger et al., 2014), and the lateral inhibition among MSNs is decreased or even disrupted (Taverna et al., 2008).

Here, we estimated the stimulus response for one particular size of the external stimulation (i.e., the number of stimulated neurons and their spatial distribution) and varied only the magnitude of the stimulation current. The size of the external stimulus may also influence the stimulus-response magnitude and reliability. We expect that increasing the number of neurons in a fixed region will increase the response reliability and magnitude. However, increasing the stimulated region with a fixed number of neurons may have a nonmonotonic effect on the response magnitude and reliability. As long as the stimulated region is smaller than the size of an individual bump, the response magnitude and reliability will increase. Increasing the size of the stimulated region beyond the single bump will recruit surrounding neurons, which should be inhibited by the bump itself, and hence, the stimulus response may decrease. In another scenario, distributing the stimulated neurons in small islands may have nonlinear effects on the response dynamics. A detailed analysis of the relationship between stimulus size and response dynamics is a complex topic and should be addressed in a separate study.

Despite the similarities and interesting insights we noted regarding the striatal activity in the healthy and in Parkinson’s disease states, we note that our model is highly simplified and ignores several key features of the striatal network. Specifically, it would be necessary to study how other components of the striatal network [e.g., interneurons, separation of the striatal network into D1- and D2-type medium spiny neuron populations (Taverna et al., 2008; Bahuguna et al., 2015), neuromodulators] affect the stability and stimulus responses in the AI and TA states. In addition, we have used only simple LIF model neurons in our networks. Voltage-dependent ion channels may introduce nonlinear effects and change the network dynamics qualitatively (Wickens et al., 1995; Rinzel, 1998). Hence, in future work, it will be important to understand how the three dynamical states identified in our study are possibly altered when neurons are endowed with voltage-dependent ionic currents or larger heterogeneity in neuron/synapse properties.

### Model validation

Recent experimental data show that the ongoing activity of the striatum can be either in an AI state or a TA state, depending on the experimental conditions (Gage et al., 2010; Adler et al., 2012; Barbera et al., 2016). Furthermore, these data also suggest that the striatum exhibits a TA-like state as animals get engaged in a task. In addition to direct measurement of NAs using modern imaging tools (Barbera et al., 2016), our model suggests that even the relationship between the firing rate and spike time irregularity (Fig. 2) can provide further indirect evidence for the existence of NAs in the striatum.

**Figure 6.**
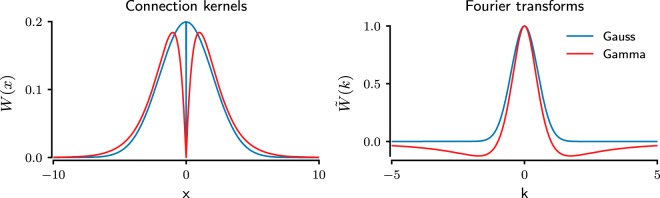
Analytical description of connectivity profiles. The graphs show the spatial connectivity profile (left) and its Fourier transform (right) as a function of the distance between neurons and wave numbers, respectively, for both gamma (red) and Gaussian (blue) connectivity kernels. Note that the spatial connectivity profile remains positive for both connectivity kernels. However, their Fourier transforms behave differently: the Gaussian kernel remains positive, whereas the gamma kernel takes negative values for larger (absolute) wave numbers.

The key to the emergence of spatially compact transient NAs in the TA state is the nonmonotonically shaped connection probability. Indirect estimates of the anatomic (from neuron morphologies) and functional connectivity both indicate that MSNs do not inhibit their nearest neighbors and that the connection probability peaks at a distance of ≈80 µm and then decays to zero beyond ≈200 µm (Fujiyama et al., 2011; López-Huerta et al., 2013). Similar estimates regarding the distance-dependent connectivity between MSNs have been drawn from computational analysis of the 3D morphologies of MSN axons and dendrites (Humphries et al., 2010). However, more experimental work is required to measure the spatial profile of not just the functional, but also the structural, connectivity within the striatum, in particular to measure the spatial connection profile and spatially compact neural clusters that would support our network model. In addition, our model predicts that neurons participating in the NAs should have a low connection probability and share their inputs. In addition, our model predicts that the *CV_ISI_* values of MSNs should increase as their firing rate increases (Fig. 2*b*). Finally, we predict that the pairwise correlation spectrum should be more susceptible to weak inputs in the TA state than in the AI state (Fig. 6). High-density sampling of striatal neurons, when available, would be sufficient to check these predictions.

**Figure 7.**
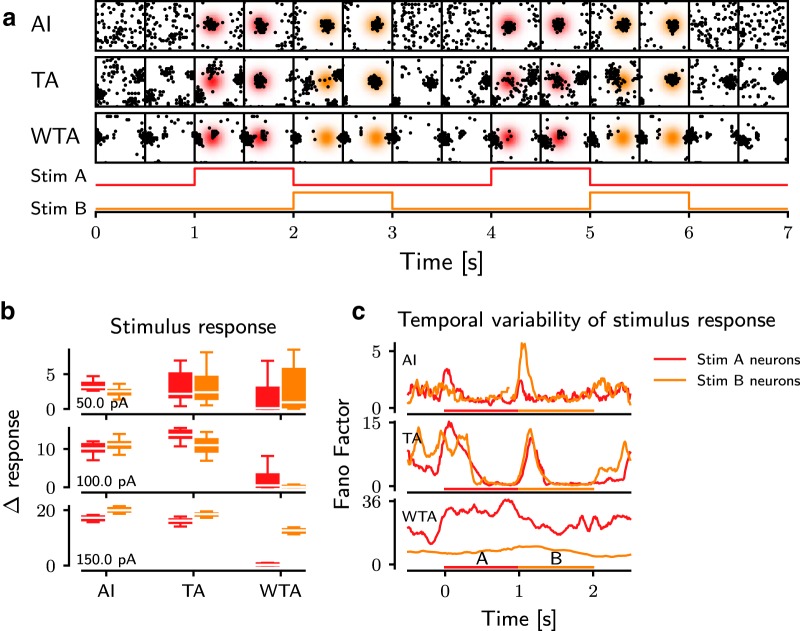
Impact of the network dynamics on the stimulus response. ***a***, Spatial distribution of the spiking activity displayed in time series for different dynamic states (rows). Each frame shows a spatial map of 30 × 30 neurons from the ROI (black squares) in a time window of 100 ms, with 500-ms intervals between successive frames. The gradient background is the probability area for stimulated neurons, and its color refers to a stimulus phase. ***b***, The change of response activity of the stimulated neurons to their corresponding stimuli A (red) or B (orange) in different ongoing bump states (AI, TA, and WTA). Each row represents the strength of the stimuli (50–150 pA). A lower Δresponse indicates a weaker impact of the external stimuli on the network activity, and lower variance of activity reflects a higher reliability of the response. For each subpanel, the white lines are the median value of the data. The colored boxes extend from the 25% to 75% of the data, i.e., the box contains ≈50% of the data. Whiskers extend from minimum to maximum values of the data. ***c***, The temporal variability (FF) of the response of the stimulated neurons as a function of time. A lower FF indicates a higher reliability of the stimulus response. A higher FF is observed at each stimulus onset, in both the AI and TA states. In contrast, in the WTA state, the network is not able to reliably respond to external stimuli. Both stimulus phases are displayed at the bottom of each subpanel.

**Figure 8.**
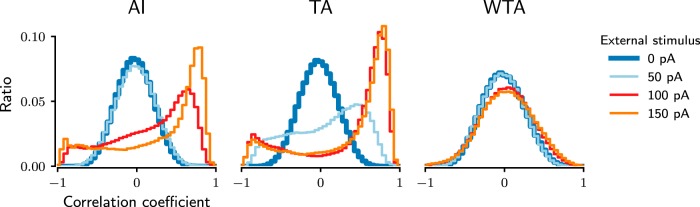
Impact of the network dynamics on the modulation of the spectrum of pairwise correlations. Different subfigures show the pairwise correlation spectrum of the network activity in three dynamic network states (AI, TA, and WTA). Different colored traces represent different stimulus strengths on the selected neurons. Compared with the correlation spectrum in ongoing activity (blue), a higher excitation is required to modulate correlations in the AI state than in the TA state. With a stronger external excitation, the correlations are more widely distributed in the network activity in both AI and TA states.

### Relationship with models of cortical networks

Recurrent networks with both excitatory and inhibitory (E-I) neurons, interconnected according to a monotonically decaying (e.g., Gaussian) connectivity profile for excitation and inhibition, can be tuned to exhibit spatially clustered or stationary bump type activity (Ben-Yishai et al., 1995; Roxin et al., 2005). A key feature of E-I networks that show spatial clusters is that the excitatory connectivity decays more rapidly with distance than the inhibitory connectivity. In such networks, the summation of excitatory and inhibitory connectivity kernels or excitatory and inhibitory synaptic strengths yield the well-known Mexican hat profile as the effective connectivity kernel with its characteristic, nonmonotonic shape. With such a connectivity profile, local recurrent excitation activates neighboring neurons which, in turn, inhibit the surrounding region because of the stronger distal inhibition. That is, in such E-I networks, a coactivated local group of neurons is brought together by their mutual, predominantly excitatory, connections and by their common field of surround inhibition. Examples of such behavior have been reported in the experimental literature, e.g., in the monkey prefrontal cortex (Vaadia et al., 1995). In contrast, we found that for purely inhibitory networks, using both network simulations and neural field equations, a nonmonotonic spatial connectivity kernel (such as the gamma distribution) generates spatially clustered bump type activity for high background input. As shown by our mean field analysis, purely inhibitory networks with a Gaussian spatial connectivity profile cannot possibly support any spatially periodic bump activity. Intuitively, that is because coactivation of neighboring neurons requires that these neurons do not inhibit each other while creating an inhibitory surround. Thus, in purely inhibitory networks, a coactivated local group of neurons is defined by the lack of mutual connectivity and the presence of a common range of surround inhibition. Mathematically, as we have shown, the key condition to have spatial clusters of activity is that the effective connectivity kernel has a nonmonotonic shape as a function of distance in the network (Fig. 6 and “Necessary conditions for the emergence of bump states”). Both the Mexican hat–shaped effective connectivity kernel of E-I networks and the gamma distribution shaped connectivity kernel of purely inhibitory networks fulfill that condition. We note that the stable bump state (WTA) observed in our network models closely resembles the grid patterns observed in the medial entorhinal cortex of rodents and that computational models of grid cells also use a nonmonotonic kernel to form connections between inhibitory neurons (Couey et al., 2013; Roudi and Moser, 2014).

In summary, we have shown how the shape of the distance-dependent recurrent connectivity profile and the strength of ongoing external background excitation together determine the state of the ongoing network activity as well as the stimulus-response properties in a purely inhibitory network, such as the striatum. These results, when properly adapted to the specific inhibitory network of interest, could provide important new insights into the functional characterization of the activity dynamics in inhibitory brain networks such as the striatum, globus pallidus, and central amygdala.

## Synthesis

Reviewing Editor: Gustavo Deco, Universitat Pompeu Fabra Decisions are customarily a result of the Reviewing Editor and the peer reviewers coming together and discussing their recommendations until a consensus is reached. When revisions are invited, a fact-based synthesis statement explaining their decision and outlining what is needed to prepare a revision will be listed below. The following reviewer(s) agreed to reveal their identity: Mark Humphries

Reviewer 1:

This paper use an inhibitory network model, likened to the striatum, to address two main questions: 1. how do different forms of distance-dependent connectivity shape the spontaneous network activity? 2. how do task-related stimuli modify this ongoing activity? To answer these questions, the authors compare monotonic (Gaussian) and non-monotonic (Gamma) distance-dependent probabilities of connections between neurons. The main result is that a Gamma distance-dependent connectivity profile exhibits three main states (AI,TA,WTA) characterized by irregular, transiently stable and stable dynamics, respectively. Consequently, task-related inputs can easily modify the irregular (AI) state, but not the WTA state. In contrast, a Gaussian connectivity profile only results in irregular activity over the network (AI state).

The questions addressed are intrinsically interesting ones from the perspective of exploring the potential repertoire of dynamics in the striatum. And some of the results certainly advance our knowledge of this repertoire. At its core, there is a solid piece of computational modelling work here, which deserves publication. However, not in the current manuscript, which has a range of serious flaws. The interpretation of which dynamical states are relevant to the striatum is confused. Some of the authors' strongest claims are demonstrably wrong from their own results. And there is extensive speculation based on little evidence from the modelling. We elaborate on these points below.

Major comments:

(1) A major claim of the paper (in the abstract, Author Summary, and elsewhere) is that the Transition Activity (TA) state, of transient spatially localised activity bumps, most closely resembles the activity in striatum. It clearly does not. The model is explored under conditions of uniform (in rate and number) background input to every single neuron. The TA state appears in a particular range of this uniform input. The paper then compares these transient bumps - now called assemblies - to the evidence of striatal assemblies in the papers of Adler et al (2013) and Barbera et al (2016). But these are assemblies evoked by tasks, and time-locked to behaviour. They are, by definition, not spontaneous activity.

Indeed, the experimentally-observed assemblies are perfectly well explained as the recruitment of small groups of striatal neurons by task-related cortical input. This is exactly the case in the paper's own Fig 7A, where clustered groups are recruited by external input for the AI state.

Indeed, even by the authors' own criterion - of understanding what the downstream GP nucleus is reading out from striatum - it makes no sense that the TA state is the default state of the striatum. For if spontaneous assemblies form, how can the downstream nucleus tell the difference between those and the evoked assemblies? Why would spontaneous assemblies not then cause aberrant action selection by disinhibiting the GP's targets?

Consequently, we would argue that the paper's results are stronger evidence for the exact opposite conclusion: the AI state is the spontaneous state of the striatum, within which assemblies can be recruited by task-specific input. Both these fit the existing data on in vivo striatal activity during rest and task episodes. And anything that causes increased excitability of the striatum, pushing it into the TA or in extremis the WTA state, is then deleterious.

(2) A major claim of the paper is that the WTA state is deleterious because in this state external inputs cannot affect the ongoing activity (e.g pg 7:“in the WTA state... the external stimuli proved insufficient to alter the ongoing bump pattern”). The authors' own results show this is not true.

The bottom row of Fig 7a shows very clearly that the external inputs do change the network's activity. Input A does not, because it overlaps with an existing bump. Input B does (snapshot 6): it causes a new bump (upper right corner), which is then a new stable state of the network.

The PC analysis (Fig 7b,c) does not address this claim. Fig 7b shows a plot of time-averaged PC1 vs PC2 for all baselines and inputs superimposed: we observe that those dots cannot be separated by eye into clusters for the WTA state. The text claims therefore that the inputs do not affect the network. But this analysis does not address that issue: it merely shows that the phase-space of the network is widely sampled by both baseline and input-driven activity. Rather, for the inputs to have no effect, then for each single run of the inputs (Fig 7a), the 3 dots (yellow,red,blue) should be in the same place. But as we've just discussed for Fig 7a, they patently will not be.

Similarly the stimulus-averaged PCs in Fig 7c merely show a different view that there is wide variation in the response of the network, that is obscured by averaging over PC1 for all stimuli.

The authors need to appropriately re-analyse all results from Figure 7, to quantify: when inputs do in fact change the state of the WTA network; under what conditions those changes occur in response to each input; and in which they cause a change showing effects beyond the lifetime of the input itself. They then need to draw appropriate conclusions in the Results and Discussion.

They also need to discuss how their results for the inputs will depend on the size of the input relative to the size of the bumps: as Fig 5 makes clear, the bumps will of course have different spacing according to the connectivity distribution's parameters. So the effect of the inputs will depend on whether they overlap 0,1,2,... bumps.

(3) The exact same problem can be seen in Figure 8. The authors claim that “the response in the WTA state was not repeatable.” While Figure 8 in its current state is difficult to understand (see below), this also appears to be false from the authors' own results. Figure 8c shows the correlations between activity upon repeated inputs. Tracing along either the diagonal or up the columns, we can see that, after the first input pair, (i) each input causes strong correlation within the same group of neurons and (ii) each input causes orthogonal correlation to the other group of neurons. Consequently, the WTA-state network seems to repeat its response just fine.

The authors need to appropriately re-analyse the results in Figure 8, and draw more accurate conclusions in the Results and Discussion. They also need to explain accurately what Figure 8 is showing (a problem not restricted to this figure - see list below).

(4) There are many places where there are claims of results or speculation of relevance that are far beyond what is established in the paper. Moreover, the Results are full of speculation best left to the Discussion at best, or, preferably, omitted. We name a few:

(i) Abstract and elsewhere: “the TA shows maximal variability and stimulus sensitivity”. Nowhere are these properties addressed in the paper

(ii) Abstract and elsewhere: the claim that PD is related to an increase in cortical input, so loss of dopamine causes a shift to the WTA state. What evidence is there that PD causes an increase in cortical firing rate? Even if it does, this is not evidence that the loss of dopamine causes a shift to the WTA state. The model does not consider dopamine, whose actions are, to say the least, complicated. pg 21 asserts that “the WTA state closely resembles the striatum dynamics in Parkinson's disease”, which is far too strong.

(iii) Results pg 16 and elsewhere: switching between stimulus responses is slower in the TA network, which is claimed as good because “such short-term memory of the previous stimulus could be used to encode temporal associations”. How?

(iv) Discussion pg19 and elsewhere: “...we showed the TA state... provides the necessary correlation structure to learn such NAs”. There is no evidence in the paper for how correlations in striatal activity lead to learning, let alone that these are “necessary” correlations.

Minor comments:

* pg 10-11: the paper claims here that the entropy of the population coincident spike rate (Hs) tells us something about the spatial arrangement of the activity. It does not. Indeed, the authors own Fig 4A shows that the entropy and the number of bumps do not have a monotonic relationship. Either explain what the change in entropy actually tells us, or remove entirely.

* All simulations (apart from Figure 5) seem to be from 1 Gaussian and 1 Gamma network. How then can the authors simply assert (i) the behaviour of the firing rate and regularity distributions (Fig 2), and (ii) the input rates that cause the transitions between states? (Fig 3 and middle of page 11).

* The work lacks context. There is no comparison with the most directly relevant models, which is concerning. Especially:

(i) Wickens and colleagues 1995 model (Synapse, 20, p281): simulation and analysis of a similar model of inhibitory neurons, which also uses a 2D network in a torus, showing conditions of stable bump formation when using homogenous connection patterns (uniform in all directions). Their work also goes beyond the present manuscript to show that inhomogenous connectivity would result in travelling waves, because of the torus.

(ii) Zilmer, Brunel & Hansel 2009 (Phys Rev E, 79, 031909): an extensive study of the dynamical states of an inhibitory network of LIF neurons, including the transitions between stable periodic and irregular dynamics.

(iii) Humphries et al 2010 (PLoS Comput Biol, 6, e1001011): a full model of the striatum's distance-dependent anatomy, and the influence of that connectivity on dynamics

* Limitations of the work are barely touched on. The study's relevance to the striatum, or otherwise, is not discussed. Make clear the limitations, including:

(i) The use of LIF neurons, which are far from a model of striatum medium spiny neurons: e.g. no hyperpolarisation by KIR

(ii) The lack of interneurons. Their feed-forward dynamics mean their effect on the activity states of the MSNs will clearly depend on the relative overlaps of connectivity distributions

Text comments:

* Methods:

(i) Why use only an ROI? (of 30x30). Are all measurements of bumps (e.g. Pg 11) done within a ROI?

(ii) “we measured the average of the first two PCs”. As these are plotted as points, we infer here the average over time was taken. Make this precise

* pg 9: the paragraph beginning “In Gaussian networks the firing rate distribution” repeats the previous paragraph, and could be excised without issue.

* pg9-10: the text continually refers to the Gamma network firing rate distribution as “widening” and being “wider” than the Gaussian one. Not true: the distributions are equally wide; the Gamma network's firing rate distribution is more skewed. Correct throughout

* pg 14 The section “Properties of the ongoing network activity, required to represent external stimuli” is (a) for the Discussion, not the Results; (b) superfluous anyway as of the two main properties, “flexible” is wrong (see point 2 of the main comments) and “elastic” - the return to baseline - is not tested at all. In addition, it is not clear why one should favour “flexible” and “elastic” as suitable for an unknown and unspecified computation. The following section on “Impact of the ongoing activity...” is simply motivated by the need to understand how inputs from external stimuli interact with the spontaneous dynamics. Removing all the references to “elastic” and “flexible” here would actually make it more powerful, easier to read, and not confuse results (the simulations) with the interpretation.

* pg 19. “whether MSN-MSN synapses exhibit... STDP (Fino and Venance 2010)”. There is no work on MSN-MSN STDP. The cited review, and all of the work by Venance, Fino and colleagues is on cortico-striatal STDP.

* pg. 19 “what feature of basal ganglia performs such an off-line clustering of spiking activity into assemblies...” This statement does not follow from the preceding discussion. The prior models observed correlated activity of small groups of neurons, in small networks representing a tiny speck of the striatum. So all neurons in that speck would project to a small, overlapping set of neurons in the target structures. The quoted problem would only be a problem if someone had built a network the size of the whole striatum, and found assemblies formed by cells across the entire thing, which could not then plausibly project to the same target neurons.

* pg 23. A long discussion of EI networks, which is of little relevance here. Cut.

* Many issues with the Figures and their legends:

Fig 1: what are the numbers (1K...) above each raster plot?

Figure 4:

(i)panel a: why is distance a percentage? What is the mean taken over: all neurons in 1 network at this distance, or an average over networks?

(ii) panel b: not mentioned in the text

(iii) panel c: what are the error bars for the bump count taken over?

(iv) panel d: what does (rel) mean? Proportion?

(v) panel e: how are they three classes of bumps (transient, unstable, persistent) defined?

Figure 7:

panel b: how are the solid lines defined?

Legend: “population activity in PCA” makes no sense

Figure 8:

Our reading was that: (i) the columns show correlations for repeats of the same stimulus pair; (ii) each column is a different stimulus pair. Each block of correlations is between neurons. The questions here include:

(i) What neurons are being correlated? Just those directly receiving input A (labelled A on the x-axis) and those receiving input B (labelled B on the x-axis)?

(ii) What are the correlations shown between inputs - why are they not also blocks of the same size if they are between the same groups of neurons?

(iii) What are the matrices II-IV showing? The legend mentions on-diagonal and off-diagonal averages, but these are labelled “within” and “between”: we instead thought “within” was the column-average, and “between” the row-average.

---

Reviewer 2:

In the present article, the authors present a modeling study assessing how the pattern of spontaneous activity and response to external inputs vary within a single population of inhibitory neurons depending on the spatial profile of synaptic connection between neurons. They interpret their results in the specific context of the striatal projection neurons, the medium spiny neurons (MSN). They find that connectivity profiles that display a non-monotonic dependence on distance, with a probability of connection peeking at non-zero distance, allow the emergence of spatially inhomogeneous activity profiles with local activity bumps. Depending on the strength of the initial background input, the bumps can be barely emergent, transient and driven by external input, or strongly persistent and independent of input profile. They interpret their results in light of striatal activity pattern observed experimentally.

General comments:

The modeling study appears sound the main result of this modeling study may be interesting to interpret current and future experimental results. However, the presentation lacks clarity (in particular some figures) and the bibliography appears quite partial. Also, the previous theoretical attempting to answer similar (or maybe the same) general question on the conditions required for the emergence of an activity bump in a network including lateral inhibition are only very partially discussed and cited. Finally, the sensitivity of the model to changes in parameters is not evaluated at all, making the results potentially not so general.

Concerning the clarity of figures and results:

- Figure 2: it is difficult to compare Gaussian and Gamma networks on this figure, as the color plot of distribution are plotted against different X-axis. It's not easy to see that one is more skewed than the other, as the authors claim.

- Figure 3: the (short) legend does not allow the reader to understand how this figure was generated, and in particular the parameter that was varied to go from AI to TA to WTA. This is obviously crucial here, but we have to infer from the text that external input was varied, and we don't know by how much.

- There is apparently no ref to Figure 5 in the text... If this figure is not part of the results, it's not clear why it's shown.

- Figure 7 is very hard to read. What are the dots in a? Spikes? The text refers to a minimal current Iminstim, but it's never defined so its relation to the figure is unclear. Is it state dependent? Legends for 7b and 7c also could be easier to read and interpret. Fig 7c is supposed to show that WTA is slowest. I don't see why. It's not clear, especially if you compare TA for 100pA to WTA for 150pA.... The result therefore does not seem supported by the figure in my opinion.

- Figure 8 is not clear either: Each 'stimulus trial' seems to consist in 2 sequential stim. Is that true? And is there spontaneous activity before and after like in Fig 7 (that would explain the light ares)? It should be specified in the legend, if it's the same stimulation pattern as in 7, as no stimulation pattern is shown here, unless I missed it.

Concerning the experimental bibliography:

- The studies looking at connectivity between MSNs are only very partially cited I believe. One important study for example was Chuhma, Tanaka, Hen and Rayport (2011) reporting high connection probability between MSN using optogenetics (and contrasting with previous in vitro studies cited here). A short but more exhaustive review of the existing literature need to be done to provide estimates of connection proba and any evidence for spatial pattern of connectivity. It would help to clarify the relationship between distance and number of connected neighbours in the model and literature, to better compare the model and anatomical data.

- In the introduction, references to papers concerning the function and dysfunction of GB (go and no-go paths for ex) are extremely partial: an extensive literature exists on this topic and citing a single computational study is surprising.

- Discussion about healthy and DA-depleted has almost no experimental ref.

Concerning the theoretical literature on the topic, the authors cite Roxin et al, 20005 and some other recent papers. But the conditions underlying the appearance of an activity bump in a neuronal population including inhibition traces back at least to BenYishai, Hansel and Sompolinski, 1997. Actually, a book chapter before that (not sure where though) also explored the case of purely inhibitory networks. Again here, the literature must be adequately cited. A paragraph discussing general conditions for the emergence of an activity bump with or without excitation would be welcome in the discussion...

As the difference between IA and TA appears to be only a difference in external input, it is not really surprising that IA with strong stimulation has the same behaviors as TA with lower stimulation, no?

In general, no specific prediction is made. It would be nice to propose experimentally testable predictions of the model.

Specific comments:

INTRO

BG is plural

Striatal spontaneous activity is not 1-3, most MSNs are quite at rest (0)

”Neuronal assemblies” are not defined

It's unclear why NA should emerge necessarily from WTA or WLC

”PS state” is not defined

METHODS

Is there any heterogeneities in the network?

What is the size of simulated subset: 45 neurons?

RESULTS

P14 which synaptic weights can increase f'(nu0)??

P15: why is “elastic” better than “plastic” for function? Doesn't it depend on the network function? For working memory for example, plastic is required.

End of page 17: 'therefore, the WTA state is not suitable to encode incoming stimuli': Quite vague, as 'encoding' is not really defined....

DISCUSSION:

It is proposed that the model is “consistent with the high CV of striatal neurons”. But it is not specific to MSN: pyramidal neurons in the cortex also show very high CVs

More generally, the relation between firing rate and CV in the model seems quite unspecific. Is there a specific prediction here?

## References

[B1] Adler A, Finkes I, Katabi S, Prut Y, Bergman H (2013) Encoding by synchronization in the primate striatum. J Neurosci 33:4854–4866. 10.1523/JNEUROSCI.4791-12.2013 23486956 PMC6619016

[B2] Adler A, Katabi S, Finkes I, Israel Z, Prut Y, Bergman H (2012) Temporal convergence of dynamic cell assemblies in the striato-pallidal network. J Neurosci 32:2473–2484. 10.1523/JNEUROSCI.4830-11.2012 22396421 PMC6621802

[B3] Albin RL, Young AB, Penney JB (1989) The functional anatomy of basal ganglia disorders. Trends Neurosci 12:366–375. 10.1016/0166-2236(89)90074-X 2479133

[B4] Angulo-Garcia D, Berke JD, Torcini A (2015) Cell assembly dynamics of sparsely-connected inhibitory networks: a simple model for the collective activity of striatal projection neurons. PLoS Comput Biol 12:e1004778. 10.1371/journal.pcbi.1004778 PMC4767417 26915024

[B5] Bahuguna J, Aertsen A, Kumar A (2015) Existence and control of Go/No-Go decision transition threshold in the striatum. PLoS Comput Biol 11:e1004233. 10.1371/journal.pcbi.1004233 25910230 PMC4409064

[B6] Barbera G, Liang B, Zhang L, Gerfen CR, Culurciello E, Chen R, Li Y, Lin DT (2016) Spatially compact neural clusters in the dorsal striatum encode locomotion relevant information. Neuron 92:202–213. 10.1016/j.neuron.2016.08.037 27667003 PMC5087607

[B7] Ben-Yishai R, Bar-Or RL, Sompolinsky H (1995) Theory of orientation tuning in visual cortex. Proc Natl Acad Sci U S A 92:3844–3848. 10.1073/pnas.92.9.3844 7731993 PMC42058

[B8] Brunel N (2000) Dynamics of sparsely connected networks of excitatory and inhibitory spiking neurons. J Comput Neurosci 8:183–208. 10809012 10.1023/a:1008925309027

[B9] Brunel N, Hakim V (1999) Fast global oscillations in networks of integrate-and-fire neurons with low firing rates. Neural Comput 11:1621–1671. 10.1162/089976699300016179 10490941

[B10] Burkitt A (2006) A review of the integrate-and-fire neuron model: I. Homogeneous synaptic input. Biol Cybernet 95:1–19. 10.1007/s00422-006-0068-6 16622699

[B11] Carrillo-Reid L, Hernández-López S, Tapia D, Galarraga E, Bargas J (2011) Dopaminergic modulation of the striatal microcircuit: receptor-specific configuration of cell assemblies. J Neurosci 31:14972–14983. 10.1523/JNEUROSCI.3226-11.2011 22016530 PMC6623553

[B12] Costa RM (2011) A selectionist account of de novo action learning. Curr Opin Neurobiol 21:579–586. 10.1016/j.conb.2011.05.004 21641793

[B13] Costa RM, Lin SC, Sotnikova TD, Cyr M, Gainetdinov RR, Caron MG, Nicolelis MAL (2006) Rapid alterations in corticostriatal ensemble coordination during acute dopamine-dependent motor dysfunction. Neuron 52:359–369. 10.1016/j.neuron.2006.07.030 17046697

[B14] Couey JJ, Witoelar A, Zhang SJ, Zheng K, Ye J, Dunn B, Czajkowski R, Moser MB, Moser EI, Roudi Y, Witter MP (2013) Recurrent inhibitory circuitry as a mechanism for grid formation. Nat Neurosci 16:318–324. 10.1038/nn.3310 23334580

[B15] Ermentrout B (1992) Complex dynamics in winner-take-all neural nets with slow inhibition. Neural Netw 5:(1):415–431. 10.1016/0893-6080(92)90004-3

[B16] Fieblinger T, Graves SM, Sebel LE, Alcacer C, Plotkin JL, Gertler TS, Chan CS, Heiman M, Greengard P, Cenci MA, Surmeier DJ (2014) Cell type-specific plasticity of striatal projection neurons in parkinsonism and L-DOPA-induced dyskinesia. Nat Commun 5:5316. 10.1038/ncomms6316 25360704 PMC4431763

[B17] Fujiyama F, Sohn J, Nakano T, Furuta T, Nakamura KC, Matsuda W, Kaneko T (2011) Exclusive and common targets of neostriatofugal projections of rat striosome neurons: a single neuron-tracing study using a viral vector. Eur J Neurosci 33:668–677. 10.1111/j.1460-9568.2010.07564.x 21314848

[B18] Gage GJ, Stoetzner CR, Wiltschko AB, Berke JD (2010) Selective activation of striatal fast-spiking interneurons during choice execution. Neuron 67:466–479. 10.1016/j.neuron.2010.06.034 20696383 PMC2920892

[B19] Gewaltig MO, Diesmann M (2007) NEST (NEural Simulation Tool). Scholarpedia 2:1430. 10.4249/scholarpedia.1430

[B20] Humphries MD, Wood R, Gurney K (2009) Dopamine-modulated dynamic cell assemblies generated by the GABAergic striatal microcircuit. Neural Netw 22:1174–1188. 10.1016/j.neunet.2009.07.018 19646846

[B21] Humphries MD, Wood R, Gurney K (2010) Reconstructing the three-dimensional GABAergic microcircuit of the striatum. PLoS Comput Biol 6:e1001011. 10.1371/journal.pcbi.1001011 21124867 PMC2991252

[B22] Hutt A, Atay FM (2005) Analysis of nonlocal neural fields for both general and gamma-distributed connectivities. Phys D Nonlin Phenom 203:30–54. 10.1016/j.physd.2005.03.002

[B23] Kimchi EY, Laubach M (2009) The dorsomedial striatum reflects response bias during learning. J Neurosci 29:14891–14902. 10.1523/JNEUROSCI.4060-09.2009 19940185 PMC6666004

[B24] Kumar A, Rotter S, Aertsen A (2008a) Conditions for propagating synchronous spiking and asynchronous firing rates in a cortical network model. J Neurosci 28:5268–5280. 10.1523/JNEUROSCI.2542-07.2008 18480283 PMC6670637

[B25] Kumar A, Schrader S, Aertsen A, Rotter S (2008b) The high-conductance state of cortical networks. Neural Comput 20:1–43. 10.1162/neco.2008.20.1.1 18044999

[B26] López-Huerta VG, Carrillo-Reid L, Galarraga E, Tapia D, Fiordelisio T, Drucker-Colin R, Bargas J (2013) The balance of striatal feedback transmission is disrupted in a model of parkinsonism. J Neurosci 33:4964–4975. 10.1523/JNEUROSCI.4721-12.2013 23486967 PMC6619024

[B27] Mahon S, Deniau JM, Charpier S (2004) Corticostriatal plasticity: life after the depression. Trends Neurosci 27:460–467. 10.1016/j.tins.2004.06.010 15271493

[B28] Mallet N, Ballion B, Le Moine C, Gonon F (2006) Cortical inputs and GABA interneurons imbalance projection neurons in the striatum of parkinsonian rats. J Neurosci 26:3875–3884. 10.1523/JNEUROSCI.4439-05.2006 16597742 PMC6674115

[B29] Mehring C, Hehl U, Kubo M, Diesmann M, Aertsen A (2003) Activity dynamics and propagation of synchronous spiking in locally connected random networks. Biolog Cybernet 88:395–408. 10.1007/s00422-002-0384-4 12750902

[B30] Planert H, Szydlowski SN, Hjorth JJJ, Grillner S, Silberberg G (2010) Dynamics of synaptic transmission between fast-spiking interneurons and striatal projection neurons of the direct and indirect pathways. J Neurosci 30:3499–3507. 10.1523/JNEUROSCI.5139-09.2010 20203210 PMC6634087

[B31] Ponzi A, Wickens J (2010) Sequentially switching cell assemblies in random inhibitory networks of spiking neurons in the striatum. J Neurosci 30:5894–5911. 10.1523/JNEUROSCI.5540-09.2010 20427650 PMC6632589

[B32] Rabinovich M, Volkovskii A, Lecanda P, Huerta R, Abarbanel HD, Laurent G (2001) Dynamical encoding by networks of competing neuron groups: winnerless competition. Phys Rev Lett 87:068102. 10.1103/PhysRevLett.87.068102 11497865

[B33] Rinzel J (1998) Propagating activity patterns in large-scale inhibitory neuronal networks. Science 279:1351–1355. 10.1126/Science.279.5355.1351 9478895

[B34] Roudi Y, Moser EI (2014) Grid cells in an inhibitory network. Nat Neurosci 17:639–641. 10.1038/nn.3704 24883451

[B35] Roxin A, Brunel N, Hansel D (2005) Role of delays in shaping spatiotemporal dynamics of neuronal activity in large networks. Phys Rev Lett 94:238103. 10.1103/PhysRevLett.94.238103 16090506

[B36] Smith Y, Raju D, Nanda B, Pare JF, Galvan A, Wichmann T (2009a) The thalamostriatal systems: anatomical and functional organization in normal and parkinsonian states. Brain Res Bull 78:60–68. 10.1016/j.brainresbull.2008.08.015 18805468 PMC2656644

[B37] Smith Y, Villalba R, Raju D (2009b) Striatal spine plasticity in Parkinson’s disease: pathological or not? Parkinson Related Disord 15:S156–S161. 10.1016/S1353-8020(09)70805-3 PMC3076277 20082980

[B38] Taverna S, Ilijic E, Surmeier DJ (2008) Recurrent collateral connections of striatal medium spiny neurons are disrupted in models of Parkinson’s disease. J Neurosci 28:5504–5512. 10.1523/JNEUROSCI.5493-07.2008 18495884 PMC3235738

[B39] Tepper JM, Koós T, Wilson CJ (2004) GABAergic microcircuits in the neostriatum. Trends Neurosci 27:662–669. 10.1016/j.tins.2004.08.007 15474166

[B40] Vaadia E, Aertsen A, Nelken I (1995) Dynamics of ‘neuronal interactions’ cannot be explained by ‘neuronal transients.’ Proc R Soc B Biol Sci 261:407–410. 10.1098/rspb.1995.0167 8587882

[B41] Wall N, De La Parra M, Callaway E, Kreitzer A (2013) Differential innervation of direct- and indirect-pathway striatal projection neurons. Neuron 79:347–360. 10.1016/j.neuron.2013.05.014 23810541 PMC3729794

[B42] Wickens J, Kotter R, Alexander ME (1995) Effects of local connectivity on striatal function: simulation and analysis of a model. Synapse 29:281–298. 10.1002/syn.890200402 7482288

[B43] Yim MY, Aertsen A, Kumar A (2011) Significance of input correlations in striatal function. PLoS Comput Biol 7:e1002254. 10.1371/journal.pcbi.1002254 22125480 PMC3219620

